# Crystal structure of racemic 2-[(β-arabino­pyran­osyl)­sulfanyl]-4,6-diphenylpyridine-3-carbo­nitrile

**DOI:** 10.1107/S2056989018007284

**Published:** 2018-05-25

**Authors:** Sherif F. Hammad, Doaa M. Masoud, Galal H. Elgemeie, Peter G. Jones

**Affiliations:** a Pharmaceutical Chemistry Department, Faculty of Pharmacy, Helwan University, Cairo, Egypt; b Chemistry Department, Faculty of Science, Helwan University, Cairo, Egypt; c Institut für Anorganische und Analytische Chemie, Technische Universität Braunschweig, Hagenring 30, D-38106 Braunschweig, Germany

**Keywords:** crystal structure, arabinose, pyridine, hydrogen bond

## Abstract

In the racemic title compound, C_23_H_20_N_2_O_4_S, the sulfur atom is attached equatorially to the sugar ring with unequal S—C bonds, *viz*.: S—C_s_ = 1.808 (2) and S—C_p_ = 1.770 (2) Å (s = sugar, p = pyrid­yl). The dihedral angles between the pyridine ring and its attached phenyl groups are 42.24 (8) and 6.37 (14)°. In the crystal, a system of classical O—H⋯O and O—H⋯(O,O) hydrogen bonds links the mol­ecules to form tube-like assemblies propagating parallel to the *c*-axis direction. Weak C—H⋯N inter­actions are also observed.

## Abstract 2

In the racemic title compound, the sulfur atom is attached equatorially to the sugar ring with unequal S—C bonds. The dihedral angles between the pyridine ring and its attached phenyl groups are 42.24 (8) and 6.37 (14)°. In the crystal, a system of classical O—H⋯O and O—H⋯(O,O) hydrogen bonds links the mol­ecules to form tube-like assemblies propagating parallel to the *c-*axis direction.

## Chemical context   

In recent years, nucleoside analogues of pyrimidines and purines have been shown to be effective as chemical therapeutic agents against cancer cells (Yoshimura *et al.*, 2000 ▸; Elgemeie *et al.*, 2016 ▸, 2017*a* ▸,*b* ▸). Recently, heterocyclic thio­glycosides have been used as anti­metabolic agents in medicinal chemistry (Dinkelaar *et al.*, 2006 ▸; Kananovich *et al.*, 2014 ▸; Elgemeie & Abu-Zaied, 2017 ▸). We and others have designed new syntheses for pyridine thio­glycosides, which have shown strong cytotoxicity against various human cancer cell lines and block proliferation of various cancer cell lines (Komor *et al.*, 2012 ▸; Elgemeie *et al.*, 2015 ▸). It has also been shown that thio­glycosides involving pyridine and di­hydro­pyridine groups exert inhibitory effects on both DNA-containing viruses and inhibitors of protein glycosyl­ation (Agrawal *et al.*, 2017 ▸; Elgemeie *et al.*, 2010 ▸; Masoud *et al.*, 2017 ▸). Based on these significant biological findings and with the aim of identifying new potent chemotherapeutics as new anti­cancer agents with improved pharmacological and safety profiles, we have prepared several new non-classical thio­glycosides containing the pyridine ring.

Here we report a one-step synthesis of the pyridine-2-thio­arabinoside (**4**) by the reaction of the pyridine-2 (1*H*)-thione derivative (**1**) with 2,3,4-tri-*O*-acetyl-α-d-arabino­pyranosyl bromide (**2**). Thus, (**1**) reacted with (**2**) in KOH in acetone to give a product for which two isomeric *N*- or *S*-arabinoside structures were conceivable, corresponding to two possible modes of glycosyl­ation. The final deprotected product (see Scheme[Chem scheme1]) would then be either the pyridine-2-thio­arabinoside (**4**) or its regioisomer pyridine-2-thione-*N*-arab­inoside (**5**). Spectroscopic data cannot differentiate between these two structures.
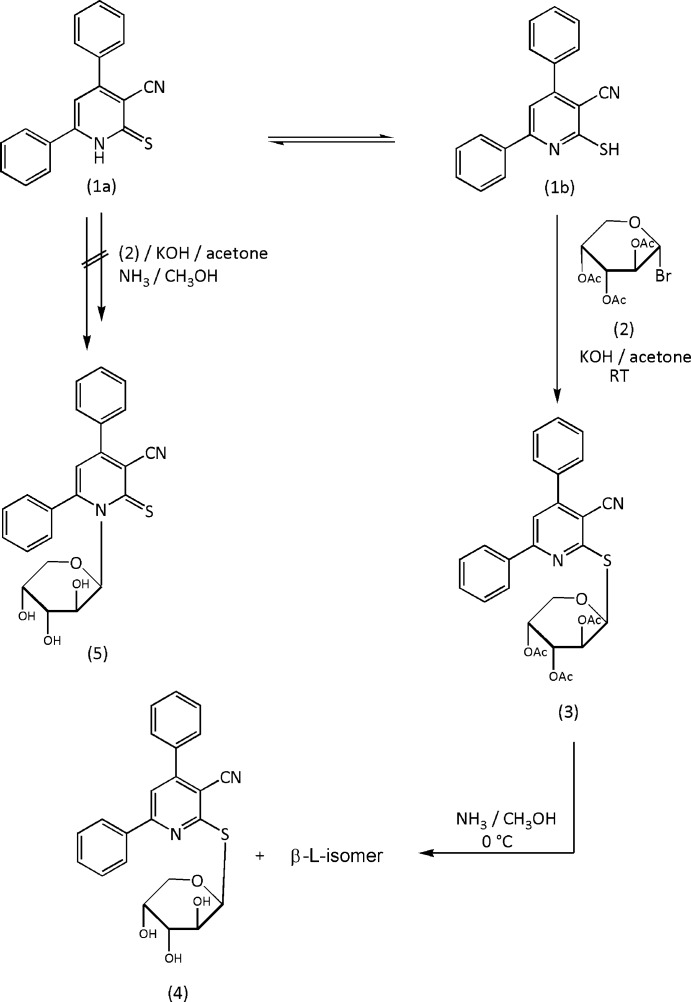


## Structural commentary   

The crystal structure determination indicated unambiguously the formation of the pyridine-2-thio­arabinoside (**4**) as the only product in the solid state. We suggest that the 2,3,4-tri-*O*-acetyl-α-d-arabinopranosyl bromide (**2**) inter­acts *via* a simple S_N_2 reaction to give the β-glycoside product (**3**), which after deprotection leads to the free 2-(β-d/l-arabino­pyran­osyl­thio)-pyridine-3-carbo­nitrile (**4**). This separates as a racemic mixture, presumably because of thermodynamic racemization during synthesis or crystallization (Brands & Davies, 2006 ▸).

**Figure 1:**
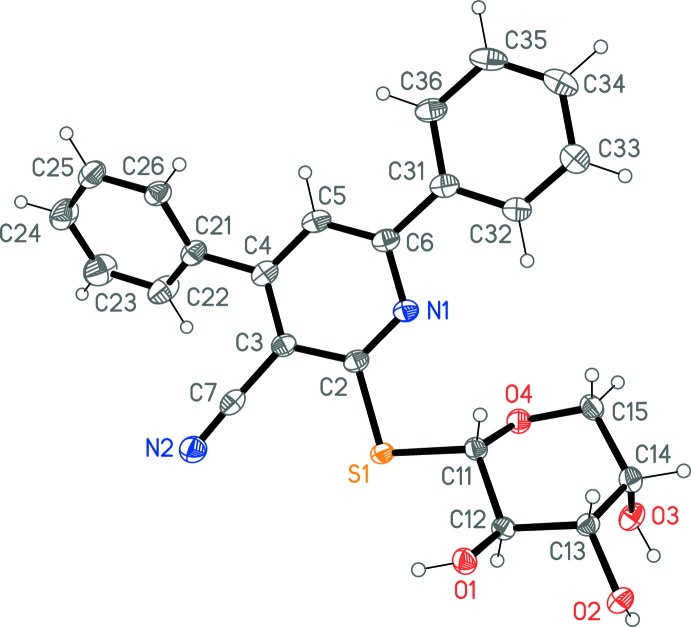
Structure of the title compound (**4**) in the crystal. Ellipsoids represent 50% probability levels.

The mol­ecular structure of (**4**) is shown in Fig. 1 ▸. The sulfur atom is attached equatorially to the sugar ring. Similarly to the structure of a related glucose derivative (Masoud *et al.*, 2017 ▸), the C—S bond lengths are unequal, with S—C_s_ 1.808 (2) and S—C_p_ 1.770 (2) Å (s = sugar, p = pyrid­yl). The phenyl ring at C31 is approximately coplanar with the pyridyl ring, but the ring at C21 is significantly rotated (inter­planar angles = 6.4 (2) and 42.24 (8)°, respectively). The relative orientation of the pyridyl ring and the sugar moiety is defined by the torsion angles N1—C2—S1—C11 9.7 (2) and C2—S1—C11—C12 162.73 (12)°. The intra­molecular contact O1—H01⋯S1, with H⋯S 2.79 (4) Å and an angle of 109 (3)°, is probably too long and has too narrow an angle to be considered a hydrogen bond.

## Supra­molecular features   

**Figure 2:**
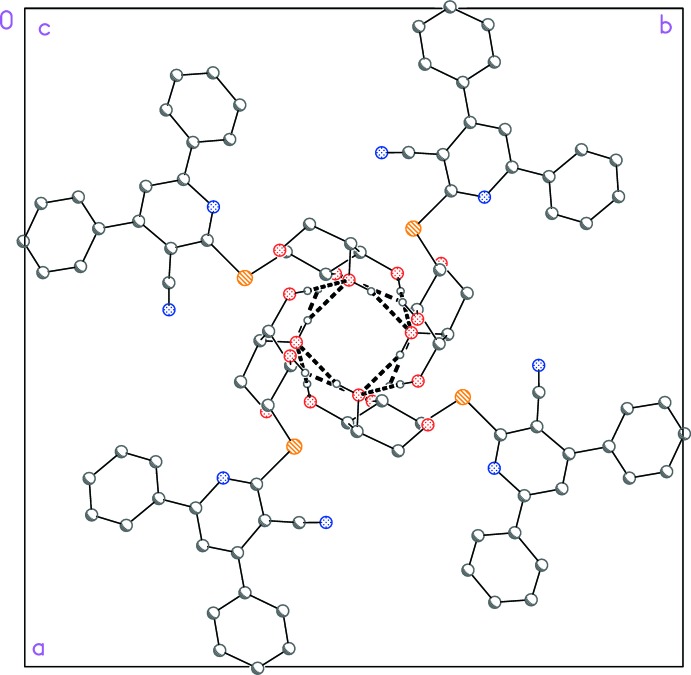
Packing diagram of (**4**) projected parallel to the *c* axis. Dashed lines indicate classical hydrogen bonds.

In the crystal, the mol­ecules are connected by two-centre O2—H02⋯O3^ii^ and O3—H03⋯O3^ii^ hydrogen bonds and a three-centre O1—H01⋯O1^i^,O2^i^ hydrogen bond (Table 1 ▸), via the 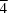 operator, thus forming tube-like assemblies parallel to the *c* axis (Figs. 2 ▸ and 3 ▸). The short S1⋯O1 (1 − *y*, *x*, 1 − *z*) contact of 3.2374 (16) Å (van der Waals’ contact distance = 3.32 Å) may play a supporting role, but is not shown explicitly.

## Database survey   

There is one other structure involving arabinose with a sulfur substituent at the C2 position; the arabinose is tri­acetyl­ated and the sulfur atom, which is axially bonded to the sugar ring, acts as a bridge to a pyran­opyrimidine ring system (Tomas *et al.*, 1993 ▸; refcode WACJAL).

## Synthesis and crystallization   

To a solution of the pyridine-2-(1*H*)-thione (**1**) (2.88 g, 0.01 mol) in aqueous potassium hydroxide (6 ml, 0.56 g, 0.01 mol) was added a solution of 2,3,4-tri-*O*-acetyl-α-d-arabino­pyranosyl bromide (**2**) (3.73 g, 0.011 mol) in acetone (30 ml). The reaction mixture was stirred at room temperature until the reaction was judged complete by TLC (30 min to 2 h). The mixture was evaporated under reduced pressure at 313 K and the residue was washed with distilled water to remove the potassium bromide. The solid was collected by filtration and crystallized from ethanol to give compound (**3**) in 70% yield (m. p. 440–442 K). Dry gaseous ammonia was then passed through a solution of the protected thio­glycoside (**3**) (0.5 g) in dry methanol (20 ml) at 273 K for 15 min, and the mixture was stirred at 273 K until the reaction was complete (TLC, 1–2 h). The mixture was evaporated at 313 K to give a solid residue, which was recrystallized from methanol solution to give compound (**4**) in 60% yield (m.p. 479–480 K), IR (KBr): 3370–3480 (OH); 2222 (CN) cm^−1. 1^H NMR (400 MHz, DMSO-*d*_6_): *δ* 3.10–3.70 (*m*, 5H, 2H-5′, H-4′, H-3′, H-2′); 4.81–5.20 (*m*, 3H, 3OH); 5.52 (*d*, 1H, H-1′), 7.05–7.78 (*m*, 10H, 2C_6_H_5_), 7.99 (*s*, 1H, pyridine H-5). Analysis calculated for C_23_H_20_N_2_O_4_S (420.47): C, 65.60%; H, 4.76%; N, 6.66%. Found: C, 65.48%; H, 4.84%; N, 6.41%.

## Refinement   

Crystal data, data collection and structure refinement details are summarized in Table 2 ▸. OH hydrogen atoms were refined freely. Other hydrogen atoms were included using a riding model starting from calculated positions (C—H_aromatic_ = 0.95, C—H_methyl­ene_ = 0.99, C—H_methine_ = 1.00 Å) with *U*_iso_(H) = 1.2–1.5*U*_eq_(C).

## Supplementary Material

Crystal structure: contains datablock(s) I, global. DOI: 10.1107/S2056989018007284/hb7743sup1.cif

Structure factors: contains datablock(s) I. DOI: 10.1107/S2056989018007284/hb7743Isup2.hkl

CCDC reference: 1843269

Additional supporting information:  crystallographic information; 3D view; checkCIF report

## Figures and Tables

**Figure 3:**
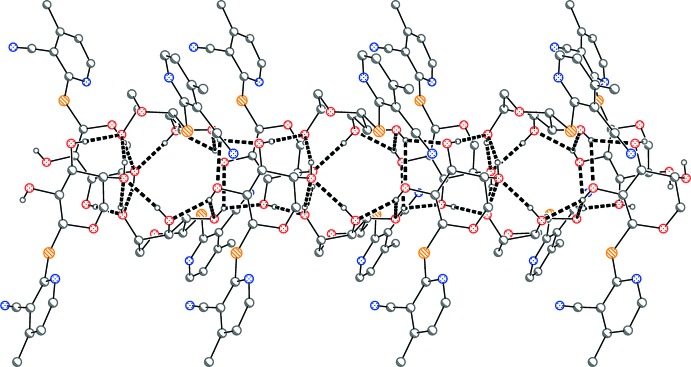
Packing diagram of (**4**) viewed parallel to the *a* axis. Dashed lines indicate classical hydrogen bonds. Phenyl rings are represented by the *ipso* carbon atoms only.

**Table 1:** Hydrogen-bond geometry (Å, °)

*D*—H⋯*A*	*D*—H	H⋯*A*	*D*⋯*A*	*D*—H⋯*A*
O1—H01⋯O2^i^	0.85 (4)	2.12 (4)	2.831 (2)	140 (3)
O1—H01⋯O1^i^	0.85 (4)	2.42 (3)	3.133 (2)	141 (3)
O2—H02⋯O3^ii^	0.81 (3)	2.07 (4)	2.883 (2)	175 (3)
O3—H03⋯O3^ii^	0.82 (4)	1.94 (4)	2.729 (2)	159 (4)
C13—H13⋯N2^iii^	1.00	2.57	3.547 (3)	165
C34—H34⋯N2^iv^	0.95	2.51	3.404 (3)	157

Symmetry codes: (i) 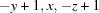; (ii) 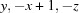; (iii) 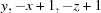; (iv) 
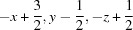
.

**Table 2:** Experimental details

Crystal data	
Chemical formula	C_23_H_20_N_2_O_4_S
*M*_r_	420.47
Crystal system, space group	Tetragonal, *P* 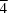 2_1_ *c*
Temperature (K)	100
*a*, *c* (Å)	21.8333 (2), 8.67551 (17)
*V* (Å^3^)	4135.54 (11)
*Z*	8
Radiation type	Cu *K*α
μ (mm^−1^)	1.67
Crystal size (mm)	0.2 × 0.2 × 0.1

Data collection	
Diffractometer	Oxford Diffraction Xcalibur, Atlas, Nova
Absorption correction	Multi-scan (*CrysAlis PRO*; Rigaku OD, 2015 ▸)
*T*_min_, *T*_max_	0.631, 1.000
No. of measured, independent and observed [*I* > 2σ(*I*)] reflections	22380, 4067, 3766
*R*_int_	0.050
(sin θ/λ)_max_ (Å^−1^)	0.629

Refinement	
*R*[*F* ^2^ > 2σ(*F* ^2^)], *wR*(*F* ^2^), *S*	0.029, 0.072, 1.04
No. of reflections	4067
No. of parameters	283
H-atom treatment	H atoms treated by a mixture of independent and constrained refinement
Δρ_max_, Δρ_min_ (e Å^−3^)	0.14, −0.21
Absolute structure	Flack *x* determined using 1455 quotients [(*I* ^+^)−(*I* ^−^)]/[(*I* ^+^)+(*I* ^−^)] (Parsons *et al.*, 2013 ▸)
Absolute structure parameter	−0.001 (9)

Computer programs: *CrysAlis PRO* (Rigaku OD, 2015 ▸), *SHELXS97* (Sheldrick, 2008 ▸), *SHELXL2017/1* (Sheldrick, 2015 ▸) and *XP* (Siemens, 1994 ▸).

## supplementary crystallographic information

### Crystal data

**Table:**

C_23_H_20_N_2_O_4_S	*D*_x_ = 1.351 Mg m^−^^3^
*M**_r_* = 420.47	Cu *K*α radiation, λ = 1.54184 Å
Tetragonal, *P*42_1_*c*	Cell parameters from 11865 reflections
*a* = 21.8333 (2) Å	θ = 4.0–75.7°
*c* = 8.67551 (17) Å	µ = 1.67 mm^−^^1^
*V* = 4135.54 (11) Å^3^	*T* = 100 K
*Z* = 8	Irregular tablet, colourless
*F*(000) = 1760	0.2 × 0.2 × 0.1 mm

### Data collection

**Table:**

Oxford Diffraction Xcalibur, Atlas, Nova diffractometer	4067 independent reflections
Radiation source: micro-focus sealed X-ray tube	3766 reflections with *I* > 2σ(*I*)
Detector resolution: 10.3543 pixels mm^-1^	*R*_int_ = 0.050
ω–scan	θ_max_ = 76.0°, θ_min_ = 4.1°
Absorption correction: multi-scan (CrysAlisPro; Rigaku OD, 2015)	*h* = −27→19
*T*_min_ = 0.631, *T*_max_ = 1.000	*k* = −23→26
22380 measured reflections	*l* = −10→10

### Refinement

**Table:**

Refinement on *F*^2^	Secondary atom site location: difference Fourier map
Least-squares matrix: full	Hydrogen site location: mixed
*R*[*F*^2^ > 2σ(*F*^2^)] = 0.029	H atoms treated by a mixture of independent and constrained refinement
*wR*(*F*^2^) = 0.072	*w* = 1/[σ^2^(*F*_o_^2^) + (0.0406*P*)^2^ + 0.206*P*] where *P* = (*F*_o_^2^ + 2*F*_c_^2^)/3
*S* = 1.04	(Δ/σ)_max_ = 0.001
4067 reflections	Δρ_max_ = 0.14 e Å^−^^3^
283 parameters	Δρ_min_ = −0.21 e Å^−^^3^
0 restraints	Absolute structure: Flack *x* determined using 1455 quotients [(*I*^+^)-(*I*^-^)]/[(*I*^+^)+(*I*^-^)] (Parsons *et al.*, 2013)
Primary atom site location: structure-invariant direct methods	Absolute structure parameter: −0.001 (9)

### Special details

**Table:**

Geometry. All esds (except the esd in the dihedral angle between two l.s. planes) are estimated using the full covariance matrix. The cell esds are taken into account individually in the estimation of esds in distances, angles and torsion angles; correlations between esds in cell parameters are only used when they are defined by crystal symmetry. An approximate (isotropic) treatment of cell esds is used for estimating esds involving l.s. planes. Least-squares planes (x,y,z in crystal coordinates) and deviations from them (* indicates atom used to define plane) 9.6921 (0.0225) x - 5.4261 (0.0258) y - 7.4689 (0.0051) z = 2.9936 (0.0259) * -0.0092 (0.0019) C21 * 0.0029 (0.0022) C22 * 0.0049 (0.0023) C23 * -0.0063 (0.0021) C24 * -0.0001 (0.0019) C25 * 0.0078 (0.0018) C26 Rms deviation of fitted atoms = 0.0060 7.1279 (0.0186) x + 10.0786 (0.0190) y - 7.1557 (0.0046) z = 5.9951 (0.0155) Angle to previous plane (with approximate esd) = 42.243 ( 0.080 ) * 0.0110 (0.0015) N1 * 0.0192 (0.0017) C2 * -0.0324 (0.0017) C3 * 0.0172 (0.0016) C4 * 0.0119 (0.0016) C5 * -0.0269 (0.0016) C6 Rms deviation of fitted atoms = 0.0212 9.0031 (0.0245) x + 8.5392 (0.0229) y - 7.1382 (0.0057) z = 7.0241 (0.0183) Angle to previous plane (with approximate esd) = 6.371 ( 0.143 ) * -0.0055 (0.0018) C31 * 0.0027 (0.0023) C32 * 0.0001 (0.0024) C33 * -0.0001 (0.0021) C34 * -0.0028 (0.0019) C35 * 0.0056 (0.0018) C36 Rms deviation of fitted atoms = 0.0036

### Fractional atomic coordinates and isotropic or equivalent isotropic displacement parameters (Å^2^)

**Table:**

	*x*	*y*	*z*	*U*_iso_*/*U*_eq_	
S1	0.66558 (2)	0.40955 (2)	0.37773 (7)	0.01892 (12)	
N1	0.71332 (8)	0.30150 (8)	0.2959 (2)	0.0193 (4)	
C2	0.72285 (9)	0.35219 (9)	0.3756 (3)	0.0188 (4)	
C3	0.77798 (10)	0.36462 (10)	0.4552 (3)	0.0196 (4)	
C4	0.82688 (10)	0.32328 (10)	0.4388 (3)	0.0213 (5)	
C5	0.81609 (10)	0.27034 (10)	0.3542 (3)	0.0228 (5)	
H5	0.847932	0.241118	0.341532	0.027*	
C6	0.75894 (10)	0.25972 (10)	0.2878 (3)	0.0206 (5)	
C7	0.78113 (10)	0.41680 (10)	0.5556 (3)	0.0216 (5)	
N2	0.78087 (9)	0.45778 (9)	0.6383 (3)	0.0282 (5)	
C11	0.60285 (9)	0.36958 (10)	0.2861 (3)	0.0180 (4)	
H11	0.600243	0.326953	0.327770	0.022*	
C12	0.54261 (10)	0.40390 (10)	0.3205 (3)	0.0175 (4)	
H12	0.545412	0.447100	0.282823	0.021*	
C13	0.49053 (9)	0.37047 (9)	0.2379 (3)	0.0174 (4)	
H13	0.485720	0.329883	0.289917	0.021*	
C14	0.50479 (10)	0.35742 (10)	0.0692 (3)	0.0205 (5)	
H14	0.472970	0.329428	0.025478	0.025*	
C15	0.56731 (10)	0.32792 (11)	0.0554 (3)	0.0228 (5)	
H15A	0.567252	0.287793	0.108559	0.027*	
H15B	0.577236	0.320966	−0.054522	0.027*	
O1	0.52797 (7)	0.40316 (7)	0.4791 (2)	0.0205 (3)	
H01	0.5536 (17)	0.4259 (16)	0.526 (5)	0.046 (10)*	
O2	0.43389 (7)	0.40239 (8)	0.2568 (2)	0.0210 (3)	
H02	0.4290 (15)	0.4296 (16)	0.194 (4)	0.035 (9)*	
O3	0.50739 (7)	0.41263 (8)	−0.0200 (2)	0.0237 (4)	
H03	0.4738 (18)	0.4298 (17)	−0.019 (5)	0.047 (10)*	
O4	0.61261 (7)	0.36736 (7)	0.1238 (2)	0.0211 (3)	
C21	0.88763 (10)	0.33451 (11)	0.5093 (3)	0.0235 (5)	
C22	0.91531 (11)	0.39202 (12)	0.5018 (4)	0.0333 (6)	
H22	0.895156	0.424849	0.450852	0.040*	
C23	0.97250 (12)	0.40132 (13)	0.5690 (4)	0.0399 (7)	
H23	0.991202	0.440544	0.563513	0.048*	
C24	1.00232 (11)	0.35385 (13)	0.6436 (4)	0.0348 (6)	
H24	1.041042	0.360614	0.690570	0.042*	
C25	0.97528 (11)	0.29625 (12)	0.6496 (3)	0.0288 (5)	
H25	0.995712	0.263483	0.700037	0.035*	
C26	0.91861 (10)	0.28654 (11)	0.5820 (3)	0.0235 (5)	
H26	0.900651	0.246932	0.585205	0.028*	
C31	0.74451 (11)	0.20354 (10)	0.1993 (3)	0.0222 (5)	
C32	0.68866 (11)	0.19904 (11)	0.1223 (4)	0.0328 (6)	
H32	0.660698	0.232346	0.125814	0.039*	
C33	0.67336 (13)	0.14672 (13)	0.0408 (4)	0.0388 (7)	
H33	0.634972	0.144225	−0.010425	0.047*	
C34	0.71408 (13)	0.09782 (11)	0.0337 (3)	0.0332 (6)	
H34	0.703694	0.061962	−0.022583	0.040*	
C35	0.76943 (12)	0.10168 (10)	0.1085 (3)	0.0285 (5)	
H35	0.797126	0.068164	0.104383	0.034*	
C36	0.78530 (11)	0.15421 (10)	0.1901 (3)	0.0244 (5)	
H36	0.823987	0.156588	0.239892	0.029*	

### Atomic displacement parameters (Å^2^)

**Table:**

	*U*^11^	*U*^22^	*U*^33^	*U*^12^	*U*^13^	*U*^23^
S1	0.0153 (2)	0.0177 (2)	0.0237 (3)	0.00191 (17)	−0.0005 (2)	−0.0018 (2)
N1	0.0185 (8)	0.0196 (8)	0.0198 (10)	0.0026 (7)	0.0021 (7)	0.0003 (8)
C2	0.0165 (9)	0.0198 (9)	0.0199 (11)	0.0019 (7)	0.0029 (9)	0.0025 (9)
C3	0.0193 (10)	0.0185 (10)	0.0210 (11)	0.0010 (8)	0.0007 (9)	0.0029 (9)
C4	0.0185 (10)	0.0235 (10)	0.0220 (12)	0.0010 (8)	0.0029 (9)	0.0066 (9)
C5	0.0212 (10)	0.0231 (10)	0.0243 (13)	0.0058 (8)	0.0044 (9)	0.0041 (9)
C6	0.0208 (10)	0.0208 (10)	0.0201 (13)	0.0037 (8)	0.0043 (9)	0.0035 (9)
C7	0.0167 (9)	0.0221 (11)	0.0260 (12)	0.0004 (8)	−0.0020 (9)	0.0068 (10)
N2	0.0277 (10)	0.0226 (9)	0.0343 (13)	0.0010 (7)	−0.0043 (9)	−0.0011 (10)
C11	0.0168 (9)	0.0196 (9)	0.0174 (11)	−0.0003 (8)	0.0016 (8)	−0.0019 (9)
C12	0.0182 (9)	0.0173 (9)	0.0169 (11)	−0.0005 (8)	0.0015 (8)	−0.0001 (8)
C13	0.0153 (9)	0.0173 (9)	0.0195 (11)	0.0004 (7)	0.0007 (8)	0.0002 (8)
C14	0.0206 (10)	0.0211 (10)	0.0200 (12)	−0.0030 (8)	0.0000 (9)	−0.0012 (9)
C15	0.0235 (10)	0.0235 (10)	0.0215 (12)	−0.0014 (9)	−0.0003 (9)	−0.0057 (9)
O1	0.0189 (7)	0.0255 (8)	0.0170 (8)	−0.0009 (6)	0.0013 (6)	−0.0033 (7)
O2	0.0159 (7)	0.0249 (8)	0.0221 (9)	0.0016 (6)	0.0008 (6)	0.0020 (7)
O3	0.0187 (7)	0.0305 (8)	0.0219 (9)	0.0003 (7)	0.0006 (7)	0.0056 (7)
O4	0.0189 (7)	0.0248 (7)	0.0196 (9)	0.0001 (6)	0.0017 (6)	−0.0023 (7)
C21	0.0191 (10)	0.0277 (11)	0.0237 (12)	0.0026 (9)	0.0010 (9)	0.0031 (10)
C22	0.0237 (11)	0.0298 (12)	0.0464 (17)	0.0009 (9)	−0.0023 (12)	0.0098 (12)
C23	0.0256 (12)	0.0336 (13)	0.061 (2)	−0.0070 (10)	−0.0021 (13)	0.0034 (14)
C24	0.0189 (10)	0.0448 (14)	0.0407 (17)	0.0004 (10)	−0.0018 (11)	0.0000 (13)
C25	0.0235 (11)	0.0362 (13)	0.0265 (14)	0.0071 (10)	−0.0009 (10)	0.0032 (11)
C26	0.0203 (10)	0.0275 (11)	0.0228 (13)	0.0045 (8)	0.0033 (9)	0.0010 (9)
C31	0.0249 (11)	0.0217 (10)	0.0201 (12)	0.0044 (9)	0.0045 (9)	0.0032 (9)
C32	0.0304 (12)	0.0273 (11)	0.0406 (16)	0.0092 (9)	−0.0058 (13)	−0.0102 (13)
C33	0.0375 (14)	0.0317 (13)	0.0473 (18)	0.0044 (11)	−0.0101 (13)	−0.0118 (13)
C34	0.0448 (14)	0.0214 (11)	0.0333 (15)	0.0011 (10)	0.0064 (12)	−0.0060 (11)
C35	0.0382 (13)	0.0172 (10)	0.0301 (14)	0.0058 (9)	0.0135 (12)	0.0045 (10)
C36	0.0262 (11)	0.0214 (11)	0.0256 (13)	0.0048 (9)	0.0059 (10)	0.0061 (10)

### Geometric parameters (Å, º)

**Table:**

S1—C2	1.770 (2)	C15—H15B	0.9900
S1—C11	1.808 (2)	O1—H01	0.85 (4)
N1—C2	1.322 (3)	O2—H02	0.81 (3)
N1—C6	1.352 (3)	O3—H03	0.82 (4)
C2—C3	1.414 (3)	C21—C22	1.395 (3)
C3—C4	1.405 (3)	C21—C26	1.397 (3)
C3—C7	1.435 (3)	C22—C23	1.393 (4)
C4—C5	1.389 (3)	C22—H22	0.9500
C4—C21	1.481 (3)	C23—C24	1.385 (4)
C5—C6	1.394 (3)	C23—H23	0.9500
C5—H5	0.9500	C24—C25	1.390 (4)
C6—C31	1.481 (3)	C24—H24	0.9500
C7—N2	1.147 (3)	C25—C26	1.385 (3)
C11—O4	1.425 (3)	C25—H25	0.9500
C11—C12	1.543 (3)	C26—H26	0.9500
C11—H11	1.0000	C31—C32	1.394 (4)
C12—O1	1.413 (3)	C31—C36	1.400 (3)
C12—C13	1.529 (3)	C32—C33	1.384 (4)
C12—H12	1.0000	C32—H32	0.9500
C13—O2	1.429 (2)	C33—C34	1.391 (4)
C13—C14	1.523 (3)	C33—H33	0.9500
C13—H13	1.0000	C34—C35	1.374 (4)
C14—O3	1.434 (3)	C34—H34	0.9500
C14—C15	1.514 (3)	C35—C36	1.392 (4)
C14—H14	1.0000	C35—H35	0.9500
C15—O4	1.439 (3)	C36—H36	0.9500
C15—H15A	0.9900		
			
C2—S1—C11	100.90 (10)	C14—C15—H15A	109.8
C2—N1—C6	118.42 (19)	O4—C15—H15B	109.8
N1—C2—C3	123.40 (19)	C14—C15—H15B	109.8
N1—C2—S1	119.13 (17)	H15A—C15—H15B	108.2
C3—C2—S1	117.41 (17)	C12—O1—H01	108 (3)
C4—C3—C2	118.3 (2)	C13—O2—H02	113 (2)
C4—C3—C7	122.3 (2)	C14—O3—H03	110 (3)
C2—C3—C7	119.30 (19)	C11—O4—C15	108.96 (17)
C5—C4—C3	117.3 (2)	C22—C21—C26	119.1 (2)
C5—C4—C21	120.5 (2)	C22—C21—C4	121.2 (2)
C3—C4—C21	122.1 (2)	C26—C21—C4	119.7 (2)
C4—C5—C6	120.6 (2)	C23—C22—C21	120.0 (2)
C4—C5—H5	119.7	C23—C22—H22	120.0
C6—C5—H5	119.7	C21—C22—H22	120.0
N1—C6—C5	121.7 (2)	C24—C23—C22	120.5 (3)
N1—C6—C31	115.4 (2)	C24—C23—H23	119.7
C5—C6—C31	122.9 (2)	C22—C23—H23	119.7
N2—C7—C3	176.7 (2)	C23—C24—C25	119.7 (2)
O4—C11—C12	109.55 (18)	C23—C24—H24	120.2
O4—C11—S1	109.72 (14)	C25—C24—H24	120.2
C12—C11—S1	109.07 (14)	C26—C25—C24	120.1 (2)
O4—C11—H11	109.5	C26—C25—H25	119.9
C12—C11—H11	109.5	C24—C25—H25	119.9
S1—C11—H11	109.5	C25—C26—C21	120.6 (2)
O1—C12—C13	106.43 (17)	C25—C26—H26	119.7
O1—C12—C11	112.04 (18)	C21—C26—H26	119.7
C13—C12—C11	108.15 (17)	C32—C31—C36	118.4 (2)
O1—C12—H12	110.0	C32—C31—C6	119.5 (2)
C13—C12—H12	110.0	C36—C31—C6	122.1 (2)
C11—C12—H12	110.0	C33—C32—C31	120.9 (2)
O2—C13—C14	112.23 (18)	C33—C32—H32	119.5
O2—C13—C12	110.90 (17)	C31—C32—H32	119.5
C14—C13—C12	112.80 (18)	C32—C33—C34	120.1 (3)
O2—C13—H13	106.8	C32—C33—H33	119.9
C14—C13—H13	106.8	C34—C33—H33	119.9
C12—C13—H13	106.8	C35—C34—C33	119.6 (2)
O3—C14—C15	106.23 (19)	C35—C34—H34	120.2
O3—C14—C13	111.67 (18)	C33—C34—H34	120.2
C15—C14—C13	109.85 (19)	C34—C35—C36	120.7 (2)
O3—C14—H14	109.7	C34—C35—H35	119.7
C15—C14—H14	109.7	C36—C35—H35	119.7
C13—C14—H14	109.7	C35—C36—C31	120.3 (2)
O4—C15—C14	109.43 (18)	C35—C36—H36	119.9
O4—C15—H15A	109.8	C31—C36—H36	119.9
			
C6—N1—C2—C3	−1.1 (4)	C12—C13—C14—C15	49.6 (2)
C6—N1—C2—S1	176.09 (17)	O3—C14—C15—O4	63.7 (2)
C11—S1—C2—N1	9.7 (2)	C13—C14—C15—O4	−57.2 (2)
C11—S1—C2—C3	−172.95 (19)	C12—C11—O4—C15	−68.6 (2)
N1—C2—C3—C4	5.2 (4)	S1—C11—O4—C15	171.64 (14)
S1—C2—C3—C4	−172.01 (18)	C14—C15—O4—C11	67.9 (2)
N1—C2—C3—C7	−171.6 (2)	C5—C4—C21—C22	136.5 (3)
S1—C2—C3—C7	11.2 (3)	C3—C4—C21—C22	−44.1 (4)
C2—C3—C4—C5	−4.8 (3)	C5—C4—C21—C26	−42.2 (3)
C7—C3—C4—C5	171.9 (2)	C3—C4—C21—C26	137.2 (3)
C2—C3—C4—C21	175.9 (2)	C26—C21—C22—C23	−1.3 (4)
C7—C3—C4—C21	−7.4 (4)	C4—C21—C22—C23	−180.0 (3)
C3—C4—C5—C6	0.7 (3)	C21—C22—C23—C24	−0.1 (5)
C21—C4—C5—C6	−180.0 (2)	C22—C23—C24—C25	0.9 (5)
C2—N1—C6—C5	−3.3 (3)	C23—C24—C25—C26	−0.5 (4)
C2—N1—C6—C31	178.8 (2)	C24—C25—C26—C21	−0.9 (4)
C4—C5—C6—N1	3.6 (4)	C22—C21—C26—C25	1.7 (4)
C4—C5—C6—C31	−178.7 (2)	C4—C21—C26—C25	−179.5 (2)
C2—S1—C11—O4	−77.26 (16)	N1—C6—C31—C32	4.8 (3)
C2—S1—C11—C12	162.73 (16)	C5—C6—C31—C32	−173.1 (3)
O4—C11—C12—O1	175.15 (17)	N1—C6—C31—C36	−175.2 (2)
S1—C11—C12—O1	−64.7 (2)	C5—C6—C31—C36	7.0 (4)
O4—C11—C12—C13	58.2 (2)	C36—C31—C32—C33	1.0 (4)
S1—C11—C12—C13	178.27 (14)	C6—C31—C32—C33	−178.9 (3)
O1—C12—C13—O2	63.3 (2)	C31—C32—C33—C34	−0.5 (5)
C11—C12—C13—O2	−176.15 (17)	C32—C33—C34—C35	0.2 (5)
O1—C12—C13—C14	−169.85 (17)	C33—C34—C35—C36	−0.5 (4)
C11—C12—C13—C14	−49.3 (2)	C34—C35—C36—C31	1.1 (4)
O2—C13—C14—O3	58.2 (2)	C32—C31—C36—C35	−1.3 (4)
C12—C13—C14—O3	−68.0 (2)	C6—C31—C36—C35	178.6 (2)
O2—C13—C14—C15	175.75 (17)		

### Hydrogen-bond geometry (Å, º)

**Table:**

*D*—H···*A*	*D*—H	H···*A*	*D*···*A*	*D*—H···*A*
O1—H01···O2^i^	0.85 (4)	2.12 (4)	2.831 (2)	140 (3)
O1—H01···O1^i^	0.85 (4)	2.42 (3)	3.133 (2)	141 (3)
O2—H02···O3^ii^	0.81 (3)	2.07 (4)	2.883 (2)	175 (3)
O3—H03···O3^ii^	0.82 (4)	1.94 (4)	2.729 (2)	159 (4)
C13—H13···N2^iii^	1.00	2.57	3.547 (3)	165
C34—H34···N2^iv^	0.95	2.51	3.404 (3)	157

Symmetry codes: (i) −*y*+1, *x*, −*z*+1; (ii) *y*, −*x*+1, −*z*; (iii) *y*, −*x*+1, −*z*+1; (iv) −*x*+3/2, *y*−1/2, −*z*+1/2.
